# Supplementary material for: Anti-VEGFR2 F(ab′)2 drug conjugate promotes renal accumulation and glomerular repair in diabetic nephropathy
Source: Nat Commun. 2023 Dec 13;14:8268. doi: 10.1038/s41467-023-43847-2 (PMC10719340; doi:10.1038/s41467-023-43847-2)
Supplement: Supplementary file 1 — Supplementary Information [file 41467_2023_43847_MOESM1_ESM.pdf]

Supplementary information for

Anti-VEGFR2 F(ab′)$_2$ drug conjugate promotes renal accumulation and glomerular repair in diabetic nephropathy

Di Liu[1#], Yanling Song[1#], Hui Chen[1], Yuchan You[1], Luwen Zhu[1], Jucong Zhang[1], Xinyi Xu[1], Jiahao Hu[1], Xiajie Huang[1], Xiaochuan Wu[1], Xiaoling Xu[2]*, Saiping Jiang[3]*, and Yongzhong Du[1, 4]*

[1]Institute of Pharmaceutics, College of Pharmaceutical Sciences, Zhejiang University, Hangzhou 310058, China.

[2]Shulan International Medical College, Zhejiang Shuren University, Hangzhou, 310015, China.

[3]Department of Pharmacy, The First Affiliated Hospital, College of Medicine, Zhejiang University, Hangzhou 310003, China.

[4]Innovation Center of Translational Pharmacy, Jinhua Institute of Zhejiang University, Jinhua 321299, China.

[#]These authors contributed equally: Di Liu, Yanling Song.

Correspondence:

Yongzhong Du, E-mail: duyongzhong@zju.edu.cn

Saiping Jinag, E-mail: j5145@zju.edu.cn

Xiaoling Xu, E-mail: ziyao1988@zju.edu.cn

Table of contents

Supplementary Fig. 1. MS data of anti-VEGFR2 F(ab′)$_2$ reduced by TCEP.

Supplementary Fig. 2. SDS-PAGE gel of IgG, IgG F(ab′)$_2$.

Supplementary Fig. 3. Synthesis of anti-VEGFR2 F(ab′)$_2$-SS31.

Supplementary Fig. 4. Western blotting and densitometric analysis of VEGFR2 expression using lysates from normal and high glucose-treated MRGECs and MPC5 cells.

Supplementary Fig. 5. Anti-VEGFR2 F(ab′)$_2$-SS31 reduced M1/M2 macrophage ratio in DN mice.

Supplementary Fig. 6. Renal cytokine IL-1β and IL-8 alternation in DN mice after different treatments.

Supplementary Fig. 7. Anti-VEGFR2 F(ab′)$_2$-SS31 inhibited fibrosis in DN mice.

Supplementary Fig. 8. ^{1}H-NMR spectra of NHS-TK-NHS.

Supplementary Table 1. The molar concentration and fluorescent intensity of Cy5 labeled agents

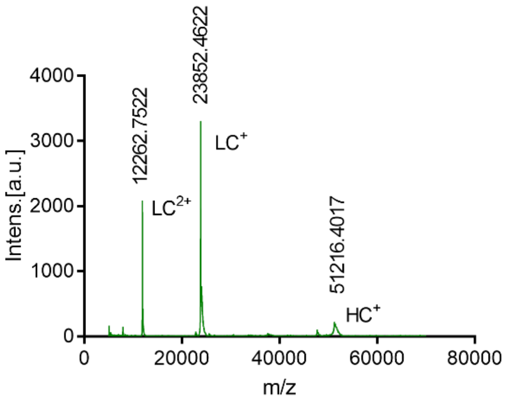

Supplementary Fig. 1. MS data of anti-VEGFR2 F(ab′)$_2$ reduced by TCEP. Detection range from 10-60 kD. LC$^+$, LC^{2+} and HC$^+$ stand for the singly protonated, singly charged light chain, the doubly protonated, doubly charged light chain, and the singly protonated, singly charged heavy chain, respectively.

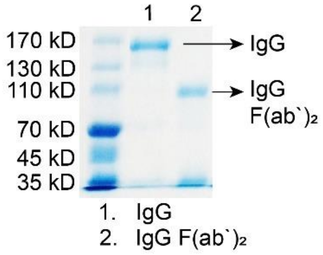

Supplementary Fig. 2. SDS-PAGE gel of IgG, IgG F(ab′)$_2$.

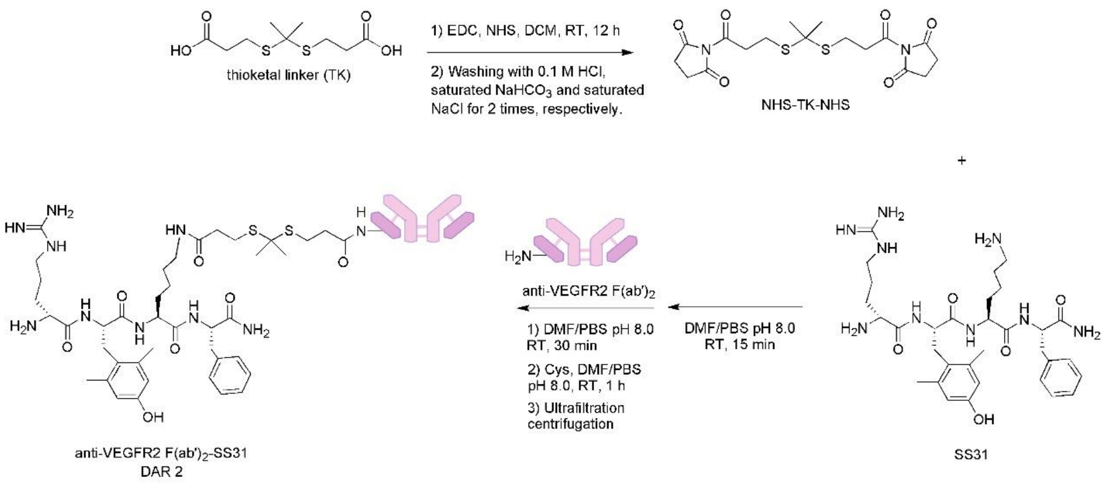

Supplementary Fig. 3. Synthesis of anti-VEGFR2 F(ab′)2-SS31.

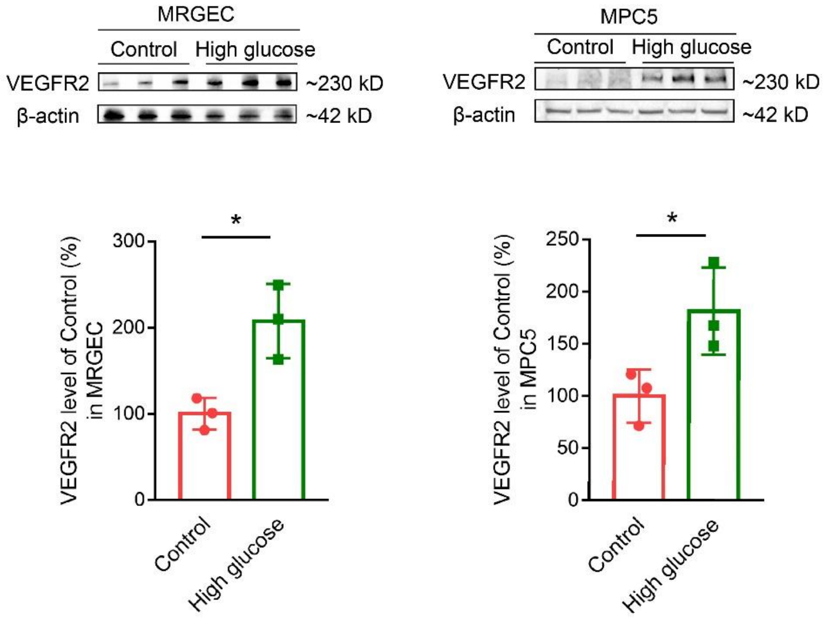

Supplementary Fig. 4. Western blotting and densitometric analysis of VEGFR2 expression using lysates from normal and high glucose-treated MRGECs and MPC5 cells. All data are expressed as mean ± s.d. Two-tailed unpaired *t* tests were used for statistical analyses. n = 3 independent experiments in each group, * $p < 0.05$. Source data are provided as a Source Data file.

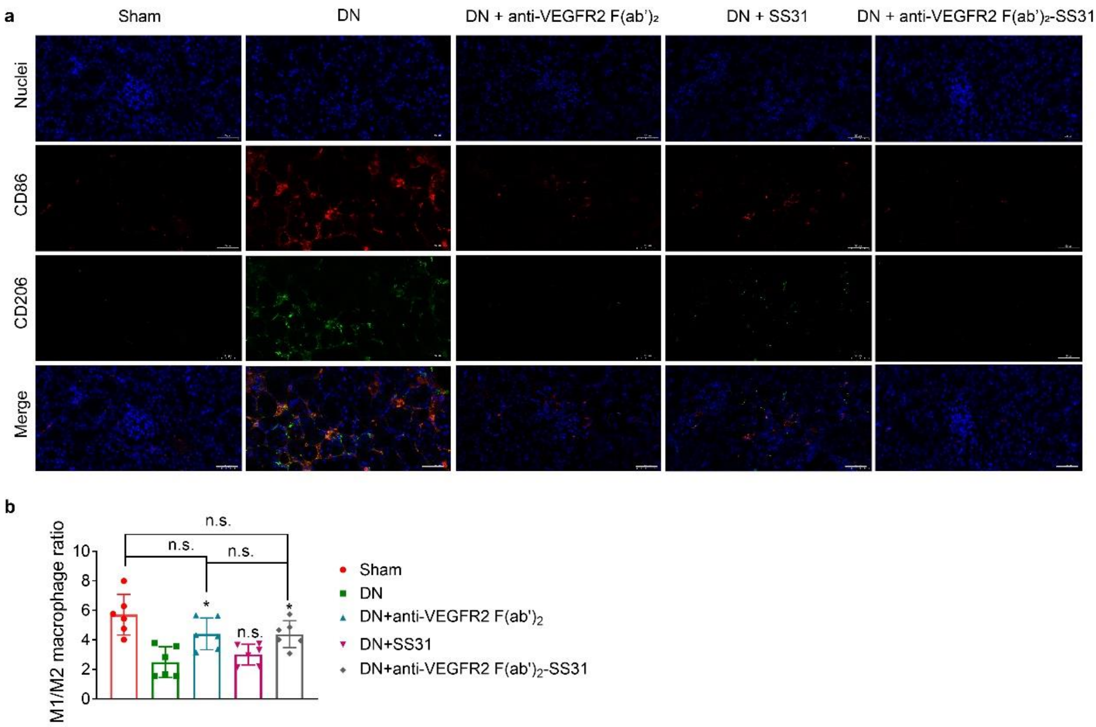

Supplementary Fig. 5. Anti-VEGFR2 F(ab′)2-SS31 reduced M1/M2 macrophage ratio in DN mice. (a) Immunofluorescence staining of CD86 and CD206 in DN kidneys after different treatments. M1 macrophages (red) are stained with CD86, and the M2 macrophages (green) are stained with CD206. Nuclei (blue), staining with DAPI. Scale bars: 50 μm. (b) The mean ratio of fluorescence intensity of M1 and M2 macrophages in (a). All data are expressed as mean ± s.d. Statistical significance was calculated using a one-way ANOVA and post-hoc test. n = 6 mice in each group, n.s. no significant difference, * $p < 0.05$ as compared with DN group. Source data are provided as a Source Data file.

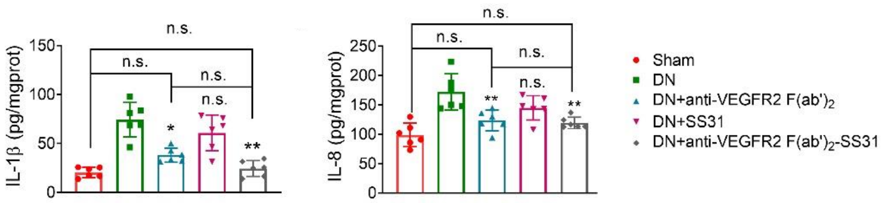

Supplementary Fig. 6. Renal cytokine IL-1β and IL-8 alternation in DN mice after different treatments. All data are expressed as mean ± s.d. Statistical significance was calculated using a one-way ANOVA and post-hoc test. n = 6 mice in each group, n.s. no significant difference, * $p < 0.05$, ** $p < 0.01$ as compared with DN group. IL-1β: interleukin-1β; IL-8: interleukin-8. Source data are provided as a Source Data file.

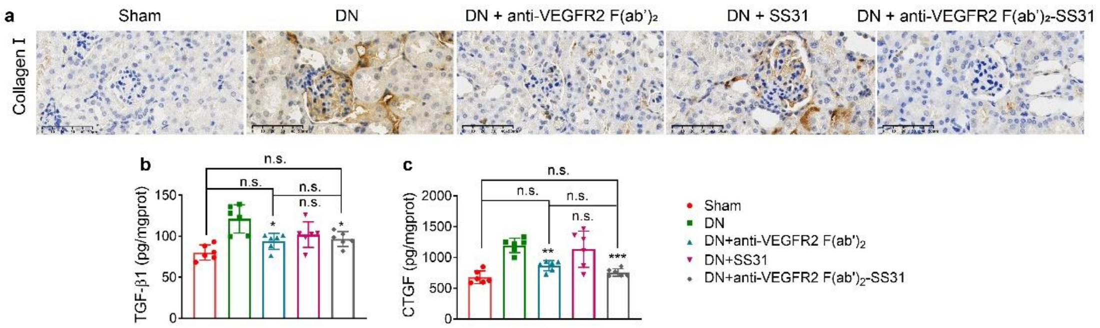

Supplementary Fig. 7. Anti-VEGFR2 F(ab′)2-SS31 inhibited fibrosis in DN mice. (a) Immunohistochemical staining (brown) of collagen I, marker of fibrosis. Scale bar, 50 μm. (b and c) The expression changes of TGF-β1 and CTGF in DN mice after different treatments. All data are expressed as mean ± s.d. Statistical significance was calculated using a one-way ANOVA and post-hoc test. n = 6 mice in each group, n.s. no significant difference, * $p < 0.05$, ** $p < 0.01$, *** $p < 0.001$ as compared with DN group. TGF-β1: transforming growth factor β1; CTGF: connective tissue growth factor. Source data are provided as a Source Data file.

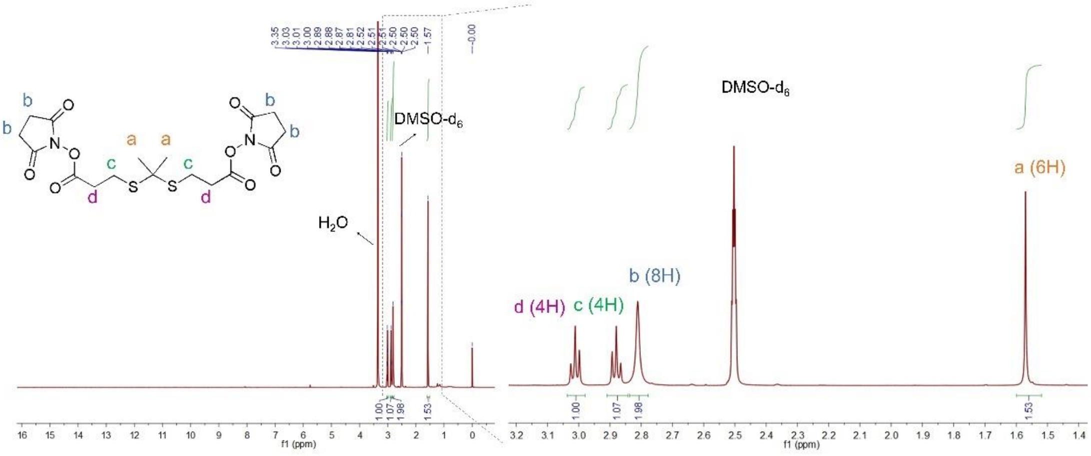

Supplementary Fig. 8. ^{1}H-NMR spectra of NHS-TK-NHS.

Supplementary Table 1. The molar concentration and fluorescent intensity of Cy5 labeled agents

Cy5 Labeled Agents	Molecular Weight (kDa)	Mass Concentration (mg/mL)	Molar Concentration (pmol/μL)	Cy5 Absorption (pmol/μL)
Isotype IgG-Cy5	~150	3.209	21.39	22.67
Isotype IgG F(ab′)2-Cy5	~110	2.318	21.07	22.56
Anti-VEGFR2-Cy5	~150	3.169	21.13	22.18
Anti-VEGFR2 F(ab′)2-Cy5	~110	2.378	21.62	22.76
Anti-VEGFR2 F(ab′)2-SS31-Cy5	~110	2.283	20.75	21.73
