# Supplementary material for: Anti-VEGFR2 F(ab′)2 drug conjugate promotes renal accumulation and glomerular repair in diabetic nephropathy
Source: Nat Commun. 2023 Dec 13;14:8268. doi: 10.1038/s41467-023-43847-2 (PMC10719340; doi:10.1038/s41467-023-43847-2)
Supplement: Supplementary file 3 — Reporting Summary [file 41467_2023_43847_MOESM3_ESM.pdf]

nature portfolio

Corresponding author(s): Yongzhong Du, Xiaoling Xu, Saiping Jiang

Last updated by author(s): Nov 10, 2023

Reporting Summary

Nature Portfolio wishes to improve the reproducibility of the work that we publish. This form provides structure for consistency and transparency in reporting. For further information on Nature Portfolio policies, see our Editorial Policies and the Editorial Policy Checklist.

Statistics

For all statistical analyses, confirm that the following items are present in the figure legend, table legend, main text, or Methods section.

n/a	Confirmed	
☐	☒	The exact sample size (*n*) for each experimental group/condition, given as a discrete number and unit of measurement
☐	☒	A statement on whether measurements were taken from distinct samples or whether the same sample was measured repeatedly
☐	☒	The statistical test(s) used AND whether they are one- or two-sided<br>*Only common tests should be described solely by name; describe more complex techniques in the Methods section.*
☒	☐	A description of all covariates tested
☒	☐	A description of any assumptions or corrections, such as tests of normality and adjustment for multiple comparisons
☐	☒	A full description of the statistical parameters including central tendency (e.g. means) or other basic estimates (e.g. regression coefficient) AND variation (e.g. standard deviation) or associated estimates of uncertainty (e.g. confidence intervals)
☐	☒	For null hypothesis testing, the test statistic (e.g. *F*, *t*, *r*) with confidence intervals, effect sizes, degrees of freedom and *P* value noted<br>*Give P values as exact values whenever suitable.*
☒	☐	For Bayesian analysis, information on the choice of priors and Markov chain Monte Carlo settings
☒	☐	For hierarchical and complex designs, identification of the appropriate level for tests and full reporting of outcomes
☒	☐	Estimates of effect sizes (e.g. Cohen's *d*, Pearson's *r*), indicating how they were calculated

Our web collection on statistics for biologists contains articles on many of the points above.

Software and code

Policy information about availability of computer code

Data collection	The mass spectra were obtained using a mass spectrometer (ultraflex TOF/TOF, Bruker, Switzerland). The NMR spectra were obtained using an NMR spectrometer (BRUKER AVIII500M, Bruker, Switzerland). The images of mice organs were collected by IVIS Spectrum imaging system (PerkinElmer, USA). Fluorescent images were collected by Leica TCS SP8 imaging system. All flow data were collected by a flow cytometer (ACEA NovoCyteTM; ACEA Biosciences). Transmission electron microscopy (TEM) images were acquired on an electron microscopy (JEOL JEM-1230, Japan).
Data analysis	The statistical analyses were conducted on Origin 8.0. All flow data were analyzed by NovoExpress1.4.1 and FlowJo(v.10). The western blot images were analyzed by Image Lab 5.2. The images of tissue section were analyzed by image J1.8.0. The fluorescent images were analyzed by MestReNova 6.1.1.

For manuscripts utilizing custom algorithms or software that are central to the research but not yet described in published literature, software must be made available to editors and reviewers. We strongly encourage code deposition in a community repository (e.g. GitHub). See the Nature Portfolio guidelines for submitting code & software for further information.

Data

Policy information about availability of data

All manuscripts must include a data availability statement. This statement should provide the following information, where applicable:

- Accession codes, unique identifiers, or web links for publicly available datasets
- A description of any restrictions on data availability
- For clinical datasets or third party data, please ensure that the statement adheres to our policy

The authors the main data supporting the results in this study are available within the paper and Supplementary Information. Source data will be provided with this paper. The raw and analyzed datasets generated during the study are too large to be publicly shared, but they are available for research purposes from the corresponding authors on reasonable request.

Research involving human participants, their data, or biological material

Policy information about studies with human participants or human data. See also policy information about sex, gender (identity/presentation), and sexual orientation and race, ethnicity and racism.

Reporting on sex and gender	*Use the terms sex (biological attribute) and gender (shaped by social and cultural circumstances) carefully in order to avoid confusing both terms. Indicate if findings apply to only one sex or gender; describe whether sex and gender were considered in study design; whether sex and/or gender was determined based on self-reporting or assigned and methods used. Provide in the source data disaggregated sex and gender data, where this information has been collected, and if consent has been obtained for sharing of individual-level data; provide overall numbers in this Reporting Summary. Please state if this information has not been collected. Report sex- and gender-based analyses where performed, justify reasons for lack of sex- and gender-based analysis.*
Reporting on race, ethnicity, or other socially relevant groupings	*Please specify the socially constructed or socially relevant categorization variable(s) used in your manuscript and explain why they were used. Please note that such variables should not be used as proxies for other socially constructed/relevant variables (for example, race or ethnicity should not be used as a proxy for socioeconomic status). Provide clear definitions of the relevant terms used, how they were provided (by the participants/respondents, the researchers, or third parties), and the method(s) used to classify people into the different categories (e.g. self-report, census or administrative data, social media data, etc.) Please provide details about how you controlled for confounding variables in your analyses.*
Population characteristics	*Describe the covariate-relevant population characteristics of the human research participants (e.g. age, genotypic information, past and current diagnosis and treatment categories). If you filled out the behavioural & social sciences study design questions and have nothing to add here, write "See above."*
Recruitment	*Describe how participants were recruited. Outline any potential self-selection bias or other biases that may be present and how these are likely to impact results.*
Ethics oversight	*Identify the organization(s) that approved the study protocol.*

Note that full information on the approval of the study protocol must also be provided in the manuscript.

Field-specific reporting

Please select the one below that is the best fit for your research. If you are not sure, read the appropriate sections before making your selection.

[x] Life sciences [ ] Behavioural & social sciences [ ] Ecological, evolutionary & environmental sciences

For a reference copy of the document with all sections, see nature.com/documents/nr-reporting-summary-flat.pdf

Life sciences study design

All studies must disclose on these points even when the disclosure is negative.

Sample size	Sample sizes were estimated on the basis of preliminary experiments and relevant literature for in vivo animal experimentation on diabetic nephropathy. Sample sizes are indicated in the Methods and Figure captions.
Data exclusions	No data were excluded.
Replication	All experiments were performed a minimum of three replicates in independent experiments with similar results. All attempts at replication
Randomization	All samples and organisms were randomly allocated into experimental groups
Blinding	For microscopy software, flow cytometry, and other data collected by objective instruments, the investigators were not blinded to group allocation during data collection because they need to know which group each raw data corresponds to. But the investigator was blinded at the time of data analysis. The laboratory personnel who performed mice experiments was not blinded because they needed to know how to

treat mice with different strategies. But the laboratory personnel was blinded during the data analysis from each individual mice.

Reporting for specific materials, systems and methods

We require information from authors about some types of materials, experimental systems and methods used in many studies. Here, indicate whether each material, system or method listed is relevant to your study. If you are not sure if a list item applies to your research, read the appropriate section before selecting a response.

Materials & experimental systems

n/a	Involved in the study	
☐	☒	Antibodies
☐	☒	Eukaryotic cell lines
☒	☐	Palaeontology and archaeology
☐	☒	Animals and other organisms
☒	☐	Clinical data
☒	☐	Dual use research of concern
☒	☐	Plants

Methods

n/a	Involved in the study	
☒	☐	ChIP-seq
☐	☒	Flow cytometry
☒	☐	MRI-based neuroimaging

Antibodies

Antibodies used

VEGFR2 primary antibody (Rat monoclonal, Bio X Cell InvivoPlus, #BP0060, Clone: DC101),
NPHS2 primary antibody (Rabbit monoclonal, Abcam, ab181143 , Clone: EPR13820),
Nitrotyrosine primary antibody (Mouse Polyclonal, GeneTex, GTX30730, Clone: 39B6),
β-actin primary antibody (Rabbit monoclonal, Proteintech, 20536-1-AP),
α-SMA primary antibody (Rabbit Polyclonal, Proteintech, 14395-1-AP),
collagen I primary antibody (Rabbit Polyclonal, Proteintech, 14695-1-AP),
F4/80 primary antibody (Rabbit monoclonal, Cell Signaling, 70076s, Clone: D2S9R),
VEGF Receptor 2 (D5B1) primary antibody (Rabbit monoclonal, Cell Signaling, 9698S, Clone: D5B1),
CD86 primary antibody (Rabbit monoclonal, Cell Signaling, 19589s, Clone: E5W6H),
CD206 primary antibody (Rabbit monoclonal, Abcam, ab300621, Clone: EPR25215-277),
FITC Goat Anti-Rabbit IgG antibody (Beyotime, A0562),
Rabbit Anti-Mouse IgG HRP antibody (Haoke, HKI0029),
Goat Anti-Rabbit IgG HRP antibody (Beyotime, A0208),
Goat Anti-Rat IgG F(ab')2 fragment HRP antibody (GeneTex, GTX26517).

Validation

No customized antibodies were used. Validation data of the antibodies purchased from commercial vendors are available on the manufactures' website and data sheets.
VEGFR2 primary antibody: https://bioxcell.com/invivoplus-anti-mouse-vegfr-2-bp0060
NPHS2 primary antibody: https://www.abcam.cn/products/primary-antibodies/nphs2-antibody-epr13820-ab181143.html
Nitrotyrosine primary antibody: https://www.genetex.cn/Product/Detail/Nitrotyrosine-antibody-39B6/GTX30730
β-actin primary antibody: https://www.ptgcn.com/products/ACTB-Antibody-20536-1-AP.htm
α-SMA primary antibody: https://www.ptgcn.com/products/ACTA2-Antibody-14395-1-AP.htm
collagen I primary antibody: https://www.ptgcn.com/products/COL1A2-Antibody-14695-1-AP.htm
F4/80 primary antibody: https://www.cellsignal.cn/products/primary-antibodies/f4-80-d2s9r-xp-rabbit-mab/70076
VEGF Receptor 2 (D5B1) primary antibody: https://www.cellsignal.cn/products/primary-antibodies/vegf-receptor-2-d5b1-rabbit-mab/9698
CD86 primary antibody: https://www.cellsignal.cn/products/primary-antibodies/cd86-e5w6h-rabbit-mab/19589
CD206 primary antibody: https://www.abcam.cn/products/primary-antibodies/mannose-receptor-antibody-epr25215-277-ab300621.html
FITC Goat Anti-Rabbit IgG antibody: https://www.beyotime.com/product/A0562.htm
Rabbit Anti-Mouse IgG HRP antibody: https://www.haokebio.com/4995.html
Goat Anti-Rabbit IgG HRP antibody: https://www.beyotime.com/product/A0208.htm
Goat Anti-Rat IgG F(ab')2 fragment HRP antibody: https://www.genetex.cn/Product/Detail/Goat-Anti-Rat-IgG-F-ab-2-antibody-F-ab-2-fragment-pre-adsorbed-HRP/GTX26517

Eukaryotic cell lines

Policy information about cell lines and Sex and Gender in Research

Cell line source(s)	Mouse renal glomerular endothelial cell line (MRGECs) were purchased from Procell Biology (Wuhan, China). Mouse podocyte clone5 (MPC5) cells were purchased from Fuheng Biology (Shanghai, China).
Authentication	STR analysis was used for cell line authentication.
Mycoplasma contamination	Both MRGECs and mpc5 cells tested negative for mycoplasma contaimination.

Commonly misidentified lines (See ICLAC register)	No commonly misidentified cell lines were used.

Animals and other research organisms

Policy information about studies involving animals; ARRIVE guidelines recommended for reporting animal research, and Sex and Gender in Research

Laboratory animals	ICR mice (male, 6 to 8 weeks old, 20-25 g) were purchased from Zhejiang Academy of Medical Sciences (Hangzhou, China). The mice were housed in conventional conditions with standard food and water. The mice were housed at approximately 22±2 degrees centigrade, humidity 50±10% on a 12 h light/ 12 h dark cycle.
Wild animals	The study did not involve wild animals.
Reporting on sex	The sex of the animals used in this study is provided in the Methods. Only male mice were used in the experiments with ICR mice.
Field-collected samples	The study did not involve samples collected from the field.
Ethics oversight	All animal experiments were performed following the National Institutes of Health Guide for the Care and Use of Laboratory Animals with the approval of the Scientific Investigation Board of Zhejiang University, Hangzhou, China, under production license number ZJU20230213.

Note that full information on the approval of the study protocol must also be provided in the manuscript.

Flow Cytometry

Plots

Confirm that:

- [x] The axis labels state the marker and fluorochrome used (e.g. CD4-FITC).
- [x] The axis scales are clearly visible. Include numbers along axes only for bottom left plot of group (a 'group' is an analysis of identical markers).
- [x] All plots are contour plots with outliers or pseudocolor plots.
- [x] A numerical value for number of cells or percentage (with statistics) is provided.

Methodology

Sample preparation	MRGECs and MPC5 cells were seeded into 12-well plates and incubated with high glucose medium for 24 h. Different samples (anti-VEGFR2 F(ab')2, SS31, or anti-VEGFR2 F(ab')2-SS31 at a concentration of 0.4 μM) were added and incubated for another 24 h. Then, cells were harvested, resuspended in the buffer, incubated with annexin V-fluorescein isothiocyanate, stained with propidium iodide (Beyotime), and examined using a flow cytometer
Instrument	ACEA NovoCyteTM (ACEA Biosciences).
Software	NovoExpress software (Agilent) and FlowJo
Cell population abundance	For the detection of apoptosis in vitro, 10,000 cells were analysed for fluorescence intensity in the defined gate.
Gating strategy	Cells were first gated by FSC-H and SSC-H, followed by FSC-H and FSC-A to obtain the single cell populations. Then the positive and negative populations with respective fluorochrome were gated.

- [x] Tick this box to confirm that a figure exemplifying the gating strategy is provided in the Supplementary Information.
